# Urges to Move and Other Motivation States for Physical Activity in Clinical and Healthy Populations: A Scoping Review Protocol

**DOI:** 10.3389/fpsyg.2022.901272

**Published:** 2022-07-11

**Authors:** Matthew A. Stults-Kolehmainen, Miguel Blacutt, John B. Bartholomew, Daniel Boullosa, Petr Janata, Brian B. Koo, Paul C. McKee, Regina Casper, Christopher J. Budnick, Todd A. Gilson, Rebekah L. Blakemore, Alberto Filgueiras, Susannah L. Williamson, Nicholas SantaBarbara, Jessica L. Barker, Fabio Amador Bueno, Jennifer Heldring, Garrett I. Ash

**Affiliations:** ^1^Digestive Health Multispecialty Clinic, Yale – New Haven Hospital, New Haven, CT, United States; ^2^Department of Biobehavioral Sciences, Teachers College, Columbia University, New York, NY, United States; ^3^Department of Kinesiology and Health Education, The University of Texas at Austin, Austin, TX, United States; ^4^Integrated Institute of Health, Federal University of Mato Grosso do Sul, Campo Grande, Brazil; ^5^Department of Psychology, University of California, Davis, Davis, CA, United States; ^6^Center for Mind and Brain, Department of Psychology, University of California, Davis, Davis, CA, United States; ^7^Sleep Medicine Laboratory, VA Connecticut Healthcare System, West Haven, CT, United States; ^8^Yale Center for Restless Legs Syndrome, Yale School of Medicine, New Haven, CT, United States; ^9^Department of Psychology and Neuroscience, Duke University, Durham, NC, United States; ^10^Center for Cognitive Neuroscience, Duke University, Durham, NC, United States; ^11^Department of Psychiatry and Behavioral Sciences, Stanford University Medical School, Stanford, CA, United States; ^12^Department of Psychology, Southern Connecticut State University, New Haven, CT, United States; ^13^Department of Kinesiology and Physical Education, Northern Illinois University, DeKalb, IL, United States; ^14^School of Physical Education, Sport and Exercise Sciences, University of Otago, Dunedin, New Zealand; ^15^Brain Health Research Centre, University of Otago, Dunedin, New Zealand; ^16^Department of Cognition and Human Development, Rio de Janeiro State University, Rio de Janeiro, Brazil; ^17^Department of Health and Kinesiology, Texas A&M University, College Station, TX, United States; ^18^Department of Exercise and Rehabilitation Sciences, Merrimack College, North Andover, MA, United States; ^19^Department of Psychiatry and Behavioral Sciences, University of Minnesota, Minneapolis, MN, United States; ^20^Connecticut Community College Nursing Program, Gateway Community College, New Haven, CT, United States; ^21^Department of Experimental Radiation Oncology, The University of Texas M. D. Anderson Cancer Center, Houston, TX, United States; ^22^Center for Pain, Research, Informatics, Medical Comorbidities and Education Center (PRIME), VA Connecticut Healthcare System, West Haven, CT, United States; ^23^Center for Medical Informatics, Yale School of Medicine, New Haven, CT, United States

**Keywords:** physical activity, motivation, motivation states, affectively charged motivation states, urge for movement, Restless Legs Syndrome, movement disorder, groove

## Abstract

Motivation for bodily movement, physical activity and exercise varies from moment to moment. These motivation states may be “affectively-charged,” ranging from instances of lower tension (e.g., desires, wants) to higher tension (e.g., cravings and urges). Currently, it is not known how often these states have been investigated in clinical populations (e.g., eating disorders, exercise dependence/addiction, Restless Legs Syndrome, diabetes, obesity) vs. healthy populations (e.g., in studies of motor control; groove in music psychology). The objective of this scoping review protocol is to quantify the literature on motivation states, to determine what topical areas are represented in investigations of clinical and healthy populations, and to discover pertinent details, such as instrumentation, terminology, theories, and conceptual models, correlates and mechanisms of action. Iterative searches of scholarly databases will take place to determine which combination of search terms (e.g., “motivation states” and “physical activity”; “desire to be physically active,” etc.) captures the greatest number of relevant results. Studies will be included if motivation states for movement (e.g., desires, urges) are specifically measured or addressed. Studies will be excluded if referring to motivation as a trait. A charting data form was developed to scan all relevant documents for later data extraction. The primary outcome is simply the extent of the literature on the topic. Results will be stratified by population/condition. This scoping review will unify a diverse literature, which may result in the creation of unique models or paradigms that can be utilized to better understand motivation for bodily movement and exercise.

## Background

Physical activity, exercise and bodily movement in general, is the product of multi-factorial processes involving cognitive, emotional and motivational factors (Rhodes et al., 2019), the latter of which has become of central interest. Importantly, one's motivation for movement likely varies in the moment (Gernigon et al., 2004), just as one's actual movement behavior varies moment-by-moment (Sartini et al., 2015). Therefore, there has been a call to understand how motivation for physical activity works *in the moment* or *right now* (Stults-Kolehmainen et al., 2020, 2021). Transient desires to move or rest are driven by at least 3 main factors: (1) the preceding set of behaviors; the desire to move increases (i.e., craving rises) with prolonged sitting and decreases with excessive activity, as recently demonstrated by Stults-Kolehmainen and colleagues (Stults-Kolehmainen et al., 2021) and others (Taylor et al., 2022), (2) anticipations of pleasure and displeasure (hedonic motivation) (Williams and Evans, 2014; Rhodes et al., 2019; Williams et al., 2019) and (3) the innate drive to move (and rest) (Feige, 1976; Rowland, 1998; Garland et al., 2011), similar to thirst or hunger, that is reinforcing in and of itself. In other words, movement relieves the tension that builds with inactivity, just as drinking relieves the tension that builds with thirst. This conceptual understanding of motivation states stands in contrast to the more conventional understanding of motivation as relating to traits (e.g., “I am not a motivated person”) or enduring characteristics of a person (e.g., “Recently, I have felt ready to exercise”), goals (e.g., “I want to gain 5 pounds of muscle this Spring”), motives (e.g., “fitness”), affective and emotional antecedents (e.g., “When I feel stressed I am not physically active”), or anticipated outcomes (e.g., “Exercise makes me feel good”) (Roberts and Treasure, 2012; Stults-Kolehmainen et al., 2013; Williams and Evans, 2014; Naves-Bittencourt et al., 2015; Hoare et al., 2017; Williams et al., 2019).

Kavanagh et al. (2005) first described the concept of *affectively-charged motivation states* (ACMS) – desires, wants, cravings and urges – as applied to health behaviors (e.g., smoking, drinking, eating snacks, etc.). Ostensibly, desires and wants are weaker motivation states while urges and cravings feel stronger, may last longer and likely have a greater impact on behavior (Desmurget and Sirigu, 2012). Importantly, ACMS involve a physiological component - physical tension felt as discomfort, which relates directly to the craving and drives behavior to relieve these states. This is clearly relevant for movement and sedentary behaviors as both excessive sitting (i.e., deprivation of activity) and excessive physical activity (i.e., deprivation of rest), whether at work or during leisure time, prompt physical discomfort and fatigue, instigating action (Stults-Kolehmainen et al., 2020, 2021). However, concerning the notion of groove (i.e., the ability of music to motivate muscular movement), the driver is typically a positive tension, such as the motivation to go dance to a funk band on a Friday night as a form of relaxation and for enjoyment (Janata et al., 2012, 2018). In both cases, the tension is internal or endogenous to the person. However, there are also exogenous drivers of desire, such as environmental conditions (e.g., lighting, images, temperature, and nature). Another example is music that is high in groove, which induces an urge to move, irrespective of a person's initial states. In other words, absent hearing the music, the motivation or urge to move might be low (no endogenous driver), but upon experiencing a sensory signal with particular qualities, the urge to move and possible movement increases substantially. Overall, one may think of motivation states as actionable feelings - perceptual lodestones that attract attention to a behavior to move or be sedentary. Recent models of behavior demonstrate how these processes may influence cognition (Hofmann and Van Dillen, 2012) or be highly automatic, incorporating affective valuations that vary by the endogenous or exogenous nature of the stimulus (Brand and Ekkekakis, 2018).

Our group recently described how motivation states apply to movement and sedentary behaviors as proposed in the WANT model (Wants and Aversions for Neuromuscular Tasks) (Stults-Kolehmainen et al., 2020). This framework describes how desires and urges to move and rest reflect a range of motivational inputs that can result in ACMS being complementary or act in opposition. In other words, they are loosely coupled and operate asymmetrically in response to various situations and a range of stimuli, from stressful circumstances to meditation. For instance, if studying late at night, one might have a strong urge to rest or sleep, and to remain seated while studying, but one also may feel antsy, and have a strong desire to get up and move. The model also specifies aversions, dread or “diswants” for movement and rest, as in the case of abstaining from movement to avoid pain (Barke et al., 2012; Glaviano et al., 2019). While the model has yet to be rigorously tested, a significant leap forward was made when an instrument was created to facilitate measurement of motivation states for movement and sedentarism – the CRAVE scale (Cravings for Rest and Volitional Energy Expenditure) (Stults-Kolehmainen et al., 2021). The CRAVE is a 13-item survey gauging ACMS with two versions: “right now” and “over the past week”.

The WANT model and subsequent work (Stults-Kolehmainen et al., 2020, 2021) ties together a previously unconnected literature describing urges to move and other affectively-charged motivation states (ACMS). Most of the literature is predominately clinical, describing ACMS when such states are (a) highly problematic and (b) central to the disorder, such as in exercise dependence/addiction (Hausenblas and Downs, 2002), Restless Legs Syndrome (Khan et al., 2017), bipolar disorders/mania (Cheniaux et al., 2014), akathisia (i.e., intense urges to move related to drug use) (Iqbal et al., 2007), tic disorder (Sanger et al., 2010) anorexia nervosa/starvation (Keys et al., 1950; Casper, 2020; Casper et al., 2020), and terminal restlessness and agitation (Brajtman, 2003). Over 50% of individuals with eating disorders report excessive movement behaviors (Fietz et al., 2014). However, ACMS have also been extensively described in motor control, in music psychology (i.e., “groove”) (Janata et al., 2012, 2018; Madison and Sioros, 2014) and, more recently, exercise psychology (Ponnada et al., 2022; Taylor et al., 2022). Examining this diverse literature, it appears that the desire or urge to move is influenced by a number of stable characteristics (e.g., age) (Stults-Kolehmainen et al., 2021), food, drugs (e.g., caffeine, amphetamines) (Kaplan et al., 1997; Ferreira et al., 2006), and medicine (e.g., haloperidol, lurasidone and vilazodone) (Tripathi et al., 2019), and situational and environmental factors (Levitin et al., 2018).

Unfortunately, there are no systematic reviews spanning the topic apart from one conceptual development paper and narrative review that did not aim to comprehensively address the topic (Stults-Kolehmainen et al., 2020). Consequently, unknowns include: (a) the scope of the literature describing ACMS, (b) the relevance of apparently-related concepts (e.g., restlessness; appetence; psychomotor retardation), and (c) the frequency of ACMS in pertinent literatures, such as diabetes (Warren et al., 2003) and depressive disorders (Buyukdura et al., 2011). One major hindrance is a lack of an appropriate search strategy to find relevant studies in scientific databases. For instance, using “motivation”, “state^*^” and “physical activity” returns ~2,750 results in Web of Science. Using “movement” instead of PA results in 1,603. This investigation aims at rectifying these issues with a scoping review.

## Review Objectives

A scoping review is employed to determine the research landscape for a topic, particularly when no previous reviews have been conducted, the literature is highly heterogeneous and quantitative methods are difficult to utilize (Munn et al., 2018). This review aims to systematically and comprehensively investigate motivation states for human bodily movement, physical activity and exercise - examining all types of relevant research from all accessible sources. This will follow the PCC model for scoping reviews (population, concepts, and context) (Tricco et al., 2018; Peters et al., 2020).

The objectives of the review are to answer the following research questions:

1) What is the extent of the evidence for motivation states for movement (e.g., desires or urges to move) in both clinical and healthy populations - *in any context*?
2) What topical areas are represented in studies incorporating clinical and/or healthy populations?
3) What nomenclature is ascribed to motivation states in these content areas (e.g., “urges,” “wants” or “desires” to move; restlessness; appetence; “drive for activity”) (Casper, 2018)?
4) What descriptors are used within the current scientific literature to describe such states (e.g., the valence and/or magnitude of the feelings)?
5) What is the theoretical basis of such states, and do conceptual models exist to explain related phenomena?
6) Is a systematic review or meta-analysis feasible at this juncture (Stults-Kolehmainen and Sinha, 2014)?

This scoping review also aims to discover other pertinent details, such as how frequently these states are measured with validated instruments, correlates and mechanisms of action that may be common to or differentiate motivation states in various populations. The data analysis will be mostly descriptive, per the guidelines from Peters (Peters et al., 2020). The current approach will include quantitative and qualitative research on various motivation states (i.e., wants, desires, urges, and cravings) for physical activity.

## Inclusion Criteria

### Types of Participants Included

Following the PCC model (Tricco et al., 2018; Peters et al., 2020), the inclusion criteria will involve all human populations in any context, as motivation states are theoretically relevant and observable across the spectrum of human experience. Included will be healthy individuals (of all ages), as well as clinical populations, such as individuals with movement disorders (e.g. Parkinson's disease, tic disorder, etc.), exercise-related disorders (i.e., dependence/addiction), etc. We may also consider animal models (Ferreira et al., 2006), such as in primates and rodents, but will not incorporate inquiries on robotics/AI.

### Types of Research Included

As this is a scoping review, all types of research will be considered, including “gray literature.” There is no specific intervention of interest. It is anticipated that the database search will find a wide variety of different investigations, including retrospective and prospective observational, case-control, cohort, cross-sectional, pilot and case studies. We expect a limited number of randomized controlled trials, cross-over trials and non-randomized intervention studies. Review articles will be considered. Only full-text, English and Portuguese-language articles will be included.

## Methods

### Outcomes

The scoping review seeks to answer six main questions (see above). The primary outcome is simply the extent of the literature on the topic of motivation states. Supplementary Material 1 provides information for how each question will be addressed. Wherever possible, information will be presented quantitatively (e.g., frequency, percentages). Note that we will not specifically search for ACMS for sedentary behavior in this review but will note any findings when doing the search for movement ACMS.

### Procedures

A three-stage process will be utilized to identify published research articles relevant to the research question. First, an initial, limited search of databases will take place in PubMED, Scopus, Web of Science, and APA PsycInfo. This will look solely within titles and index terms in order to determine feasibility of anticipated search terms, whether new ones may be needed, and which combination of search terms (e.g., “motivation states” and “physical activity”; “urge to move”; “desire to be physically active”) captures the greatest number of relevant results. Then, a full search will be conducted searching within titles, abstracts, keywords and index terms using optimal search terms from step one. Third, if new relevant key terms are identified during the previous stages, the authors will conduct new searches. Two investigators will independently scan the literature. Studies will be included if motivation states for movement (i.e., desires, wants, cravings, and urges) are specifically addressed. Studies will be excluded based on criteria below. A charting data extraction form (see Supplementary Material 2) was developed for data extraction of all relevant documents, according to guidelines set forth by Peters (Peters et al., 2020). Guidelines for reporting evidence will be followed as outlined by the PRISMA-ScR Checklist (Tricco et al., 2018).

### Search Strategy

A unique database search strategy will be employed because conducting searches with simple terms, such as (“wants” AND “physical activity”) results in returns >10,000 articles. Instead, we will use a novel combination of the following search terms in four steps.

Step 1 (basic search): (“Motiv^*^ state^*^”) AND (Exercise OR “Physical activity”).
Step 2 (advanced search) will use *infinitive phrases* (e.g., urge to exercise), such as: (Urge^*^ OR Desire^*^ OR Crav^*^ OR Want^*^ OR Drive^*^) AND (To OR For OR “To be”) AND (Move^*^ OR Exercise OR “Physical^*^ activ^*^”).
Step 3 (using *nominalization of verb*): The last combinations of search words will be: (Move^*^ OR Exercise OR “Physical^*^ activ^*^”) AND (Urge OR Desire OR Craving). “Want” will not be used in this third step (e.g., “movement want” is highly unlikely to be relevant). Note that in PubMed the strategy will be modified because the use of the ^*^ symbol is not possible.
Step 4 (known pertinent terms): Five terms known to overlap with the phenomenon of motivation states will be searched: restlessness (mental/physical), hyperkinesia, psychomotor retardation, appetence and “drive for activity” (Keys et al., 1950).

The authors decided not to use the key term “feel like” (e.g., feel like moving or exercising) in the current searches as used elsewhere (Ponnada et al., 2022).

An initial search utilizing this novel search strategy narrows down the returns in Web of Science to 921 (from 2,750). Furthermore, a quick inspection reveals that the returns are more relevant. Abstracts will be expelled from results if they fall into one of 11 exclusion criteria categories, such as motivation being described as a trait, not in the English or Portuguese languages, if describing secondary desires for movement (i.e., whereas movement is motivated by other wants), etc. See Supplementary Material 3. In the event of a disagreement between two reviewers, a third independent and impartial reviewer will resolve the discordance. The databases will be searched from inception to June, 2022.

### Data Extraction

Articles that meet the inclusion criteria for the scoping review will have data extracted using a charting data extraction form (Supplementary Material 2) based on JBI's System for the Unified Management, Assessment and Review of Information (SUMARI) (Aromataris and Munn, 2020). We anticipate a high number of relevant returns (i.e., >500 articles), mostly due to the inclusion of studies on the topic of Restless Legs Syndrome (RLS). Consequently, studies relating to RLS will be limited to the last 5 years. An author who is an expert on the topic of RLS (BK) will guide selection of studies from before that period.

Some data will be extracted by just a single independent reviewer who will search for information, such as (1) field of study, (2) clinical or non-clinical, (3) condition or pathology, (4) study design, etc. See Supplementary Material 4 for a full list. Two reviewers will search deeper in each article to search for constructs relevant to the study of motivation states and the WANT model (Stults-Kolehmainen et al., 2020), such as (5) “physical activity,” “exercise” or bodily movement, (6) theoretical orientation, (7) description of motivation states, etc. See Supplementary Material 4 for all 19 variables of interest. A third independent and impartial reviewer will resolve any disagreement between the two reviewers. If required, corresponding authors of articles will be contacted for additional information. The charting data extraction form will be piloted with 10–20 studies in the early stages to ensure extraction can proceed smoothly.

### Data Synthesis

In scoping reviews, no formal data analysis is undertaken (Tricco et al., 2018; Peters et al., 2020); therefore, this review does not aim at statistically pooling quantitative components (as in a meta-analysis). Consequently, data synthesis will follow the procedures utilized in Peters (Peters et al., 2020). This largely involves a basic descriptive analysis (e.g., frequency of topic of interest, expressed as percentages), which will be mapped in tables and graphics. We will also stratify findings by clinical vs. non-clinical populations as aggregating across conditions is problematic, and there may be biases inherent to specific pathologies. Specific literatures that feature prominently, such as Restless Legs Syndrome, will receive further stratification (Khan et al., 2017). At this time, it is not clear if a critical appraisal (i.e., assessment of methodological quality) of individually selected studies will occur, as outlined in the Preferred Reporting Items for Systematic reviews and Meta-Analyses extension for Scoping Reviews (PRISMA-ScR) Checklist (Tricco et al., 2018), as such an analysis is not required for scoping reviews, but may be helpful. In scoping reviews, critical appraisal often takes the place of evaluation of risk of bias, and such a decision will be made once the data is extracted. Findings will be presented in narrative form, and will include tables and figures to aid in data presentation.

## Discussion

This scoping review is the first of its kind to both unify and quantify the literature on motivation states for bodily movement, physical activity and exercise to determine the state of knowledge in this area for both healthy and clinical populations. A previous narrative review and conceptual analysis from Stults-Kolehmainen et al. (2020) had the aim of developing the concept of motivation states for movement as a relevant and valid construct. However, it did not attempt to quantify the material or make systematic comparisons between relevant topical areas. This scoping review protocol has been designed to fill that gap - to understand the research landscape surrounding this concept as opposed to a specific question regarding it.

The proposed search protocol has strengths, but also some delimiters (i.e., designed limits) and limitations. To capture the greatest extent of relevant literature, we selected novel search terms crafted from infinitive phrases (e.g., “urge to move,” “desire to be physically active”). These were not exhaustive, however. For instance, we did not include phrases, such as “have to move” or “need for exercise” and related terms. We chose this approach to keep the search manageable. On a different note, some topical areas are likely to dominate returns. For instance, Restless Legs Syndrome is defined by the urge to move (Khan et al., 2017), and we anticipate that this single condition will account for >50% of studies we will find. Limited resources will prevent extracting data from this entire topical area. However, one author on this team who is an expert in this area (BK) will examine the studies from 2014 and before to select ones that may be most pertinent. Also, we have topical experts in the areas of music psychology/groove (PJ), motor control and movement disorders (RB), psychiatric disorders (AF), exercise training studies (DB), exercise and sports psychology (TG, JBB), eating disorders (RC) and muscle dysmorphia (NS). It is our hope that this expertise will allow us to safeguard against any serious omissions while highlighting the most pertinent research.

There are potential downstream implications for this proposed review; both healthy and clinical populations may ultimately receive some benefit from a better understanding of the literature on motivation states for movement. First, apart from RLS, many conditions related to diminished or excessive motivation states for movement have received relatively little recognition (e.g., akathisia) or have recently emerged in the literature (e.g., muscle dysmorphia); therefore, these disorders are poorly understood (Iqbal et al., 2007; SantaBarbara et al., 2020). This scoping review may mitigate this shortcoming by helping to describe the time course, subjective experience, pathology and important mechanisms of action (e.g., neurotransmitters, hormones, psychological antecedents). For instance, there is substantial amount of MRI data that exists on the topic of groove (Janata et al., 2012, 2018), which may be applicable in other literatures, such as sport and exercise psychology. It is also reasonable to expect that this review will result in progress in evaluation, including measurement tools and scoring, interpretation, and clinical cut-offs – as well as protocols for longitudinal assessment so patterns of ACMS may be delineated. Following these advances, the role of motivation states in conditions like burnout and depression may become clearer – helping us to understand if ACMS are useful for identifying these disorders (Sandmeir et al., 2021). These hypokinetic conditions, together with the hyperkinetic disorders previously mentioned, may fall along a common motor or movement urge dysfunction spectrum (MUDS) that includes normal sensations. The collection of these might even typify a new classification of conditions - the movement urge dysfunction disorders (MUDD), which has some verisimilitude but is still speculative.

A more pressing need is to better understand the relationship between motivation states and movement behaviors, including physical activity, exercise and displacement behaviors (Mohiyeddini et al., 2015). Part of the problem is the lack of explicit explanatory models and theories that attempt to describe this association and how it operates across a variety of conditions and contexts. However, some research paradigms exist in related topical areas (Berridge, 2012) and by examining these it may be possible to develop testable and practical models which facilitate better understanding of the current topic. Behavioral models may then be expanded to incorporate important linkages to other health behaviors (e.g., sleep), emotions and cognitions, as well as describe triggers (e.g., images, music) and other antecedents, barriers (e.g., fatigue, boredom), and additional factors (Williams et al., 2019). Down the road, there may be development of algorithms to predict when ACMS result in conscious and unconscious movement choices - as well as predictive models that could be used to identify the best targets for behavioral modification efforts. These may be employed as either treatments to diminish bothersome urges and cravings or interventions and technologies to enhance tepid wants and desires. It is imaginable that all of these advances would be more feasible with the results from this scoping review.

Proximal outcomes of this review would likely vary by the research focus or perspective (e.g., clinical, public health or athletic performance), condition and/or population and the primary objectives related with each. For instance, in the case of muscle dysmorphia, exercise addiction or eating disorders, potential deliverables could be educational modules for those affected and for psychiatric professionals to raise awareness of risks. Taking this a step further, this review may lead to the creation of interventions to reduce engagement in problematic behavior and mitigate harm caused by unhealthy movement patterns. Interventions might incorporate aspects of mindfulness, such as meditation or exercises that are inherently mindful, such as martial arts (Naves-Bittencourt et al., 2015; Stults-Kolehmainen et al., 2015). For those at risk of disease and not physically active, this review may help to map out just-in-time adaptive interventions, perhaps mobile-based, to identify ideal time points for action, which we call “CRAVE moments”, and to modify the environment or lead people to environments that both: (a) produce desire-promoting stimuli for movement and (b) reveal opportunities to act on these desires (Hardeman et al., 2019; Ash et al., 2021; Liu et al., 2021). Additionally, understanding the factors that drive a person to engage in exercise, their natural experience of movement desires, and their activity preferences, could lead to flexible and personalized exercise prescriptions, leading to better exercise initiation, engagement, further adherence and less drop out. Given that exercise is considered a “polypill” for health enhancement (Fiuza-Luces et al., 2013), such initiatives should be a priority. Please, refer to Stults-Kolehmainen et al. (2020) for a more extensive discussion of implications and applications of motivation states research. By being able to understand underlying motivation for movement, particularly as it occurs within the moment, this review can collectively enable both individuals and practitioners to exercise greater volitional control over when and how movement occurs.

## Author's Note

This review was registered on August 11, 2020 with PROSPERO (National Institute for Health Research) here: https://www.crd.york.ac.uk/prospero/display_record.php?ID=CRD42020191459.

## Author Contributions

The main conceptual ideas were developed by MS-K, MB, DB, JBB, PJ, GA, and PM, in that order. The manuscript was primarily written by MS-K, MB, and DB, in that order. Testing of the protocol search strategies was carried out by MS-K and PM. The study protocol and manuscript text were further evaluated and refined by authors JBB, GA, BK, RC, CB, TG, RB, AF, SW, NS, JLB, FB, and JH, in that order. All authors provided critical feedback, reviewed, and approved the final manuscript.

## Funding

GA was supported by a fellowship from the Office of Academic Affiliations at the United States Veterans Health Administration, a Robert E. Leet and Clara Guthrie Patterson Trust Mentored Research Award, Bank of America, N.A., Trustee, and American Heart Association Grant #852679 (GA, 2021–2024).

## Conflict of Interest

The authors declare that the research was conducted in the absence of any commercial or financial relationships that could be construed as a potential conflict of interest.

## Publisher's Note

All claims expressed in this article are solely those of the authors and do not necessarily represent those of their affiliated organizations, or those of the publisher, the editors and the reviewers. Any product that may be evaluated in this article, or claim that may be made by its manufacturer, is not guaranteed or endorsed by the publisher.

## References

[B1] Aromataris E. Munn Z. (2020). JBI Manual for Evidence Synthesis. Available online at: https://synthesismanual.jbi.global (accessed December 1, 2021).

[B2] Ash G. I. Nally L. M. Stults-Kolehmainen M. A. De-Los-Santos M. Jeon S. Brandt C.. (2021). Personalized big data for type 1 diabetes exercise support. SportRxiv[Preprint]. 10.31236/osf.io/34vdc

[B3] Barke A. Baudewig J. Schmidt-Samoa C. Dechent P. Kröner-Herwig B. (2012). Neural correlates of fear of movement in high and low fear-avoidant chronic low back pain patients: an event-related fMRI study. Pain 153, 540–552. 10.1016/j.pain.2011.11.012 22230805

[B4] Berridge K. C.. (2012). From prediction error to incentive salience: mesolimbic computation of reward motivation. Eur. J. Neurosci. 35, 1124–1143. 10.1111/j.1460-9568.2012.07990.x 22487042 PMC3325516

[B5] Brajtman S.. (2003). The impact on the family of terminal restlessness and its management. Palliat Med. 17, 454–460. 10.1191/0960327103pm779oa 12882264

[B6] Brand R. Ekkekakis P. (2018). Affective–Reflective theory of physical inactivity and exercise. Ger. J. Exerc. Sport Res. 48, 48–58. 10.1007/s12662-017-0477-9

[B7] Buyukdura J. S. McClintock S. M. Croarkin P. E. (2011). Psychomotor retardation in depression: biological underpinnings, measurement, and treatment. Prog. Neuropsychopharmacol. Biol. Psychiatry. 35, 395–409. 10.1016/j.pnpbp.2010.10.019 21044654 PMC3646325

[B8] Casper R.. (2018). Not the function of eating, but spontaneous activity and energy expenditure, reflected in “Restlessness” and a “Drive for Activity” appear to be dysregulated in anorexia nervosa: treatment Implications. Front. Psychol. 9, 2303. 10.3389/fpsyg.2018.02303 30532724 PMC6265370

[B9] Casper R.. (2020). Might starvation-induced adaptations in muscle mass, muscle morphology and muscle function contribute to the increased urge for movement and to spontaneous physical activity in anorexia nervosa? Nutrients 12, 2060 10.3390/nu12072060 32664448 PMC7400818

[B10] Casper R. Voderholzer U. Naab S. Schlegl S. (2020). Increased urge for movement, physical and mental restlessness, fundamental symptoms of restricting anorexia nervosa? Brain Behav. 10, e01556. 10.1002/brb3.1556 PMC7066368 32017454

[B11] Cheniaux E. Filgueiras A. Silva Rde A. Silveira L. A. Nunes A. L. Landeira-Fernandez J. (2014). Increased energy/activity, not mood changes, is the core feature of mania. J. Affect. Disord. 152–154, 256–261. 10.1016/j.jad.2013.09.021 24140225

[B12] Desmurget M. Sirigu A. (2012). Conscious motor intention emerges in the inferior parietal lobule. Curr. Opin. Neurobiol. 22, 1004–1011. 10.1016/j.conb.2012.06.006 22939569

[B13] Feige K.. (1976). Wesen und problematik der sportmotivation. Sportunterricht 5, 4–7.

[B14] Ferreira A. Lamarque S. Boyer P. Perez-Diaz F. Jouvent R. Cohen-Salmon C. (2006). Spontaneous appetence for wheel-running: a model of dependency on physical activity in rat. Eur. Psychiat. 21, 580–588. 10.1016/j.eurpsy.2005.02.003 17161285

[B15] Fietz M. Touyz S. Hay P. (2014). A risk profile of compulsive exercise in adolescents with an eating disorder: a systematic review. Adv. Eat. Disord. 2, 241–263. 10.1080/21662630.2014.894470

[B16] Fiuza-Luces C. Garatachea N. Berger N. A. Lucia A. (2013). Exercise is the real polypill. Physiology 28, 330–358. 10.1152/physiol.00019.2013 23997192

[B17] Garland T. Jr. Schutz H. Chappell M. A. Keeney B. K. Meek T. H. Copes L. E.. (2011). The biological control of voluntary exercise, spontaneous physical activity and daily energy expenditure in relation to obesity: human and rodent perspectives. J. Exp. Biol. 214, 206–29. 10.1242/jeb.048397 21177942 PMC3008631

[B18] Gernigon C. D'arripe-Longueville F. Delignières D. Ninot G. (2004). A dynamical systems perspective on goal involvement states in sport. J. Sport Exerc. Psychol. 26, 572. 10.1123/jsep.26.4.572

[B19] Glaviano N. R. Baellow A. Saliba S. (2019). Elevated fear avoidance affects lower extremity strength and squatting kinematics in women with patellofemoral pain. Athl. Train. Andamp Sports Health Care. 11, 192–200. 10.3928/19425864-20181029-01

[B20] Hardeman W. Houghton J. Lane K. Jones A. Naughton F. (2019). A systematic review of just-in-time adaptive interventions (JITAIs) to promote physical activity. Int. J. Behav. Nutr. Phys. Act. 16, 31. 10.1186/s12966-019-0792-7 PMC6448257 30943983

[B21] Hausenblas H. A. Downs D. S. (2002). Exercise dependence: a systematic review. Psychol. Sport Exerc. 3, 89–123. 10.1016/S1469-0292(00)00015-7

[B22] Hoare E. Stavreski B. Jennings G. L. Kingwell B. A. (2017). Exploring motivation and barriers to physical activity among active and inactive Australian adults. Sports 5, 47. 10.3390/sports5030047 PMC5968958 29910407

[B23] Hofmann W. Van Dillen L. (2012). Desire: the new hot spot in self-control research. Curr. Dir. Psychol. Sci. 21, 317–322. 10.1177/0963721412453587

[B24] Iqbal N. Lambert T. Masand P. (2007). Akathisia: problem of history or concern of today. CNS Spectr. 12, 1–13. 10.1017/S1092852900026201 17805218

[B25] Janata P. Peterson J. Ngan C. Keum B. Whiteside H. Ran S. (2018). Psychological and musical factors underlying engagement with unfamiliar music. Music Percept. 36, 175–200. 10.1525/mp.2018.36.2.175

[B26] Janata P. Tomic S. T. Haberman J. M. (2012). Sensorimotor coupling in music and the psychology of the groove. J. Exp. Psycholo. Gen. 141, 54–75. 10.1037/a0024208 21767048

[B27] Kaplan G. B. Greenblatt D. J. Ehrenberg B. L. Goddard J. E. Cotreau M. M. Harmatz J. S.. (1997). Dose-dependent pharmacokinetics and psychomotor effects of caffeine in humans. J. Clin. Pharmacol. 37, 693–703. 10.1002/j.1552-4604.1997.tb04356.x 9378841

[B28] Kavanagh D. J. Andrade J. May J. (2005). Imaginary relish and exquisite torture: the elaborated intrusion theory of desire. Psychol. Rev. 112, 446–467. 10.1037/0033-295X.112.2.446 15783293

[B29] Keys A. Brožek J. Henschel A. Mickelsen O. Taylor H. L. Simonson E.. (1950). The Biology of Human Starvation, Vol. II. Mineapolis, MN: University of Minnesota Press. 10.5749/j.ctv9b2tqv

[B30] Khan F. H. Ahlberg C. D. Chow C. A. Shah D. R. Koo B. B. (2017). Iron, dopamine, genetics, and hormones in the pathophysiology of restless legs syndrome. J. Neurol. 264, 1634–1641. 10.1007/s00415-017-8431-1 28236139

[B31] Levitin D. J. Grahn J. A. London J. (2018). The psychology of music: rhythm and Movement. Annu. Rev. Psychol. 69, 51–75. 10.1146/annurev-psych-122216-011740 29035690

[B32] Liu J. Spakowicz D. J. Ash G. I. Hoyd R. Ahluwalia R. Zhang A.. (2021). Bayesian structural time series for biomedical sensor data: a flexible modeling framework for evaluating interventions. PLOS Comput. Biol. 17, e1009303. 10.1371/journal.pcbi.1009303 PMC8412351 34424894

[B33] Madison G. Sioros G. (2014). What musicians do to induce the sensation of groove in simple and complex melodies, and how listeners perceive it. Front. Psychol. 5, 894. 10.3389/fpsyg.2014.00894 25191286 PMC4137755

[B34] Mohiyeddini C. Bauer S. Semple S. (2015). Neuroticism and stress: the role of displacement behavior. Anxiety Stress Coping, 28, 391–407. 10.1080/10615806.2014.1000878 25599405

[B35] Munn Z. Peters M. D. J. Stern C. Tufanaru C. McArthur A. Aromataris E. (2018). Systematic review or scoping review? Guidance for authors when choosing between a systematic or scoping review approach. BMC Med. Res. Methodol. 18, 143. 10.1186/s12874-018-0611-x PMC6245623 30453902

[B36] Naves-Bittencourt W. Mendonáa-de-Sousa A. Stults-Kolehmainen M. A. Fontes E. B. Cûrdova C. U. Demarzo M. M. P.. (2015). Martial arts: mindful exercise to combat stress. Eur. J. Hum. Mov. 34, 34–51.

[B37] Peters M. D. J. Marnie C. Tricco A. C. Pollock D. Munn Z. Alexander L.. (2020). Updated methodological guidance for the conduct of scoping reviews. JBI Evid. Synth. 18, 2119–2126. 10.11124/JBIES-20-00167 33038124

[B38] Ponnada A. Wang S. Chu D. Do B. Dunton G. Intille S. (2022). Intensive longitudinal data collection using microinteraction ecological momentary assessment: pilot and preliminary results. JMIR Form Res. 6, e32772. 10.2196/32772 PMC8867293 35138253

[B39] Rhodes R. E. McEwan D. Rebar A. L. (2019). Theories of physical activity behaviour change: a history and synthesis of approaches. Psychol. Sport. Exerc. 42, 100–109. 10.1016/j.psychsport.2018.11.010

[B40] Roberts G. C. Treasure D. (2012). Advances in Motivation in Sport and Exercise. Champaign, IL: Human Kinetics. 10.5040/9781492595182

[B41] Rowland T. W.. (1998). The biological basis of physical activity. Med. Sci. Sports Exerc. 30, 392–399. 10.1097/00005768-199803000-00009 9526885

[B42] Sandmeir A. Schoenherr D. Altmann U. Nikendei C. Schauenburg H. Dinger U. (2021). Depression severity is related to less gross body movement: a motion energy analysis. Psychopathology 54, 106–112. 10.1159/000512959 33647901

[B43] Sanger T. D. Chen D. Fehlings D. L. Hallett M. Lang A. E. Mink J. W.. (2010). Definition and classification of hyperkinetic movements in childhood. Mov. Disord. 25, 1538–1549. 10.1002/mds.23088 20589866 PMC2929378

[B44] SantaBarbara N. J. Nosrat S. Whitworth J. W. Ciccolo J. T. (2020). Acute psychological effects of resistance exercise in men with symptoms of muscle dysmorphia: a pilot study. J. Strength Cond. Res. 10.1519/JSC.0000000000003615 32332260

[B45] Sartini C. Wannamethee S. G. Iliffe S. Morris R. W. Ash S. Lennon L.. (2015). Diurnal patterns of objectively measured physical activity and sedentary behaviour in older men. BMC Public Health 15, 609–609. 10.1186/s12889-015-1976-y 26141209 PMC4490705

[B46] Stults-Kolehmainen M. Malcolm L. Diloreto J. Gunnet-Shoval K. Rathbun E. (2015). Psychological interventions for weight management: a primer for the allied health professional. ACSM Health Fitness J. 19, 16–22. 10.1249/FIT.0000000000000150

[B47] Stults-Kolehmainen M. A. Blacutt M. Bartholomew J. Gilson T. Ash G. McKee P.. (2020). Motivation states for physical activity and sedentary behavior: desire, urge, wanting, and craving. Front. Psychol. 11, 568390. 10.3389/fpsyg.2020.568390 PMC7677192 33240154

[B48] Stults-Kolehmainen M. A. Blacutt M. Fogelman N. Gilson T. A. Stanforth P. R. Divin A. L.. (2021). Measurement of motivation states for physical activity and sedentary behavior: development and validation of the CRAVE scale. Front. Psychol. 12, 568286. 10.3389/fpsyg.2021.568286 PMC8027339 33841225

[B49] Stults-Kolehmainen M. A. Ciccolo J. T. Bartholomew J. B. Seifert J. Portman R. S. (2013). Age and gender-related changes in exercise motivation among highly active individuals. Athletic Insight. 5, 45–63.

[B50] Stults-Kolehmainen M. A. Sinha R. (2014). The effects of stress on physical activity and exercise. Sports Med. 44, 81–121. 10.1007/s40279-013-0090-5 24030837 PMC3894304

[B51] Taylor I. M. Whiteley S. Ferguson R. A. (2022). Disturbance of desire-goal motivational dynamics during different exercise intensity domains. Scand. J. Med. Sci. Sports, 32, 798–806. 10.1111/sms.14129 35037710 PMC9305115

[B52] Tricco A. C. Lillie E. Zarin W. O'brien K. K. Colquhoun H. Levac D.. (2018). PRISMA extension for scoping reviews (PRISMA-ScR): checklist and explanation. Ann. Intern. Med. 169, 467–473. 10.7326/M18-0850 30178033

[B53] Tripathi R. Reich S. G. Scorr L. Guardiani E. Factor S. A. (2019). Lurasidone-induced tardive syndrome. Mov. Disord. Clin. Pract. 6, 601–604. 10.1002/mdc3.12812 31538095 PMC6749798

[B54] Warren R. E. Deary I. J. Frier B. M. (2003). The symptoms of hyperglycaemia in people with insulin-treated diabetes: classification using principal components analysis. Diabetes Metab. Res. Rev. 19, 408–414. 10.1002/dmrr.396 12951649

[B55] Williams D. M. Evans D. R. (2014). Current emotion research in health behavior science. Emotion Review. 6, 277–287. 10.1177/1754073914523052 PMC12975116 41815188

[B56] Williams D. M. Rhodes R. E. Conner M. T. (2019). Conceptualizing and intervening on affective determinants of health behaviour. Psychol. Health. 34, 1267–1281. 10.1080/08870446.2019.1675659 31617431

