# Supplementary material for: Urges to Move and Other Motivation States for Physical Activity in Clinical and Healthy Populations: A Scoping Review Protocol
Source: Front Psychol. 2022 Jul 11;13:901272. doi: 10.3389/fpsyg.2022.901272 (PMC9311496; doi:10.3389/fpsyg.2022.901272)
Supplement: Supplementary file 1 [file Table_1.docx]

| **Research questions and aims** | | **Quantitative and Qualitative Outcomes** |
| --- | --- | --- |
| 1 | Extent of the evidence | Number of articles  Time span (years from first to most recent product)  Types of studies (e.g., qualitative, randomized controlled trials, philosophical investigations, etc.) |
| 2 | Topical areas | Areas of literature/fields of interest represented (e.g., sports medicine, psychiatry, musicology)  Populations studied (including all human populations, rodents, primates)  Primary topic of interest (e.g., restless legs syndrome, exercise addiction/dependence, akathisia)  Clinical or non-clinical  Specifically: “physical activity”, “exercise” or bodily movement |
| 3 | Nomenclature | Motivation states: urge, want, desire, craving  Other related concepts  Main outcome measures |
| 4 | Descriptors | List of terms and descriptors found  Refers to the subjective experience of urge, want, desire, craving (ACMS)  Narrative synthesis* |
| 5 | Theoretical basis | Provision of theory or model – yes / no  List of theories and models that specifically examine motivation states, such as dual process models (e.g., Brand & Ekkekakis, 2018) or theories of emotion (e.g., Williams, Rhodes & Connor, 2019). |
| 6 | Feasibility of further analysis | Homogeneity in studies and outcomes (rendering data extraction and comparison meaningful)  Narrative synthesis*  Consensus |
| Other | Other pertinent details | See data extraction section in manuscript and Supplement 2. |
| * This question will also be answered through a narrative synthesis, where the results of the scoping review (data extraction) will be used to synthesize how the desires and urges to move have been described in the literature. | | |

**Supplement 1. Outcomes for each research question**
