# Supplementary material for: Urges to Move and Other Motivation States for Physical Activity in Clinical and Healthy Populations: A Scoping Review Protocol
Source: Front Psychol. 2022 Jul 11;13:901272. doi: 10.3389/fpsyg.2022.901272 (PMC9311496; doi:10.3389/fpsyg.2022.901272)
Supplement: Supplementary file 2 [file Table_2.docx]

| **Entry Step** | Step 1a | | Step 1b | | | | Step 2 | | | | | | | | |
| --- | --- | --- | --- | --- | --- | --- | --- | --- | --- | --- | --- | --- | --- | --- | --- |
| **Entry method** | Auto-populated | | Auto-populated | | | | One person will extract this data (usually from abstract, first and last page of article) | | | | | | | | |
| **Variable of interest** | *Search terms*  *"want to move", "urge to move" etc.* | Database  (WOS, PM or Scopus) | Author | Year | Title | | Field of study | Country of corresponding author | Human or animal | Clinical or "healthy" population | Age group | Gender /sex | Phenomenon of interest (or Condition/ pathology) | Study design | Whole body or specific body part (e.g., legs) |
| **Example** | "want to move" | Web of Science | Budnick | 2020 | Effects of… | | Sports medicine | USA | Human | Clinical | Adult | All | Physical inactivity | Cross-sectional | Whole body |
|  | | | | | | | | | | | | | | | |
| **Entry Step** | Step 3 | | | | | | | | | | | | | | |
| **Entry method** | Two people will independently extract information | | | | | | | | | | | | | | |
| **Variable of interest** | How many studies/datasets within article | Exogenous (MUSIC, chemical, environmental, supplement) or endogenous | Bodily movement, physical activity or exercise | Main outcomes measures | Specific psychological scale/instrument included for movement desires/motivation states  (YES or NO, plus information) | Article describes subjective experience of urge, want, desire, craving (ACMS)  (YES or NO) | | Theoretical orientation /conceptual model  (YES or NO, plus information) | Descriptors /Terminology used to describe the state (e.g., If and how it is felt: Positive or negative tension; magnitude) | Aversions, dread or “diswants” are measured or discussed  (YES or NO) | | Motivation states for rest/sedentary as well  (YES or NO) | | Correlates of the motivation state (done by category)  May include: Demographic, neurological, cognitive, affective, etc. | |
| **Example** | 1 | Endogenous | Bodily movement | Desires to move the body | Yes  CRAVE Scale (Stults-Kolehmainen et al., 2021) | Yes | | Yes: WANT model from Stults-Kolehmainen et al. (2020) | Negative tension | Yes | | No | | Affective | |

**Supplement 2. Data extraction charting form**
