# Supplementary material for: Urges to Move and Other Motivation States for Physical Activity in Clinical and Healthy Populations: A Scoping Review Protocol
Source: Front Psychol. 2022 Jul 11;13:901272. doi: 10.3389/fpsyg.2022.901272 (PMC9311496; doi:10.3389/fpsyg.2022.901272)
Supplement: Supplementary file 3 [file Table_3.docx]

**Supplement 3. Abstract Exclusion Criteria**

Abstracts will not be considered for analysis if they fall into one of these 11 categories:

1) relate to psychological traits and not states (unless the trait clearly refers to a series of states),

2) refer to conditional applications of the motivation state (e.g., “want to exercise *alone*…”),

3) the search terms are used as: i) an expression of words, ii) a linguistic flourish, iii) a generality or an abstract use of words rather than a concrete construct (e.g., “politicians are urged to move on the new Act….”), iv) text in past participle structure (e.g., “urged to exercise”), v) terms are used as verbs and not nouns,

4) not related to movement of the physical body (i.e., related to movement of objects).

5) come from the fields of robotics and engineering (anticipated to be common) or other tangential fields, such as manufacturing engineering, oil/energy science.

6) relate to secondary desires for movement (e.g., moving to accomplish something else, like to get a glass of water) and not bodily movement as the primary concern

7) relate to migration or “moving away” from a location

8) relate to bowel movement urgency and/or urinary incontinence

9) motivation state[s] is simply a side note and is not clearly related to the main point of paper

10) English or Portuguese language is unclear in its use of the terms

11) there is an obvious error in a search return – such as an abstract that clearly does not incorporate the search terms or is otherwise clearly irrelevant.
