# Supplementary material for: Urges to Move and Other Motivation States for Physical Activity in Clinical and Healthy Populations: A Scoping Review Protocol
Source: Front Psychol. 2022 Jul 11;13:901272. doi: 10.3389/fpsyg.2022.901272 (PMC9311496; doi:10.3389/fpsyg.2022.901272)
Supplement: Supplementary file 4 [file Table_4.docx]

**Supplement 4. Data to be extracted from relevant studies (as seen in data extraction charting form - refer to Supplement 2)**

| Extraction and personnel | Number | Factor |
| --- | --- | --- |
| Extracted by a single independent reviewer | 1 | Field of study (e.g., sports medicine, cancer) |
|  | 2 | Country of corresponding author |
|  | 3 | Participant type (i.e., human, rodent or primate) |
|  | 4 | Clinical or “healthy” |
|  | 5 | Age group (e.g., children, adults, older adults) |
|  | 6 | Gender |
|  | 7 | Phenomenon of interest or condition/pathology/topic (e.g., restless leg syndrome, exercise addiction / dependence, akathisia, musical groove; depression) |
|  | 8 | Study design (e.g., cross-sectional, case study, RCT) |
|  | 9 | Type of bodily movement described (i.e., single body part movement, whole body movement) |
| Extracted by two reviewers, who will search deeper in each article for the following constructs | 10 | Motivation state (ACMS*) instigated by endogenous or exogenous factors (e.g., internal drive state, music) |
|  | 11 | Specifically: “physical activity”, “exercise” or bodily movement |
|  | 12 | Main outcomes measures |
|  | 13 | Inclusion of a specific scale/instrument for movement desires/ motivation states |
|  | 14 | Describes the subjective experience of urge, want, desire, craving (ACMS) |
|  | 15 | Provision of theoretical orientation and/or conceptual model related to motivation states for movement |
|  | 16 | Description of motivation state as being positive, negative or neutral tension (valence described); Magnitude described |
|  | 17 | Whether aversions, dread or “diswants” are measured |
|  | 18 | Whether there is emphasis on motivation states for rest/sedentarism |
|  | 19 | Important correlates of motivation states |

* ACMS – Affectively-charged motivation states
